# Supplementary material for: Factors associated with time to return to horse racing following a clavicle fracture in jockeys competing in Great Britain: A review and analysis of medical records
Source: PLoS One. 2025 Jan 23;20(1):e0317724. doi: 10.1371/journal.pone.0317724 (PMC11756748; doi:10.1371/journal.pone.0317724)
Supplement: S1 Table — Note: * = Grand mean centred and scaled. Ref = reference or referent group. (DOCX) [file pone.0317724.s001.docx]

|  | Univariable Model | | | |  | Multivariable Model | | | | |
| --- | --- | --- | --- | --- | --- | --- | --- | --- | --- | --- |
| Independent variable | Log β | Lower 95% CI | Upper 95% CI | P value |  | Log β | Lower 95% CI | Upper 95% CI | P value |  |
| Sex |  |  |  |  |  |  |  |  |  |  |
| Male | -0.176 | -0.050 | 0.147 | 0.285 |  | 0.148 | -0.184 | 0.481 | 0.382 |  |
| Female (Ref) | **4.077** | - | - | - |  | **4.047** | - | - | - |  |
| Licence Type |  |  |  |  |  |  |  |  |  |  |
| Amateur | -0.399 | -0.859 | 0.062 | 0.090 |  | -0.323 | -1.029 | 0.384 | 0.371 |  |
| Conditional | -0.584 | -1.064 | -0.104 | 0.017 |  | -0.420 | -1.126 | 0.285 | 0.243 |  |
| Professional | -0.478 | -0.967 | 0.011 | 0.056 |  | -0.271 | -0.973 | 0.432 | 0.451 |  |
| Apprentice (Ref) | **4.356** | - | - | - |  | **4.047** | - | - | - |  |
| Jockey Type |  |  |  |  |  |  |  |  |  |  |
| Jump | -0.368 | -0.683 | -0.054 | 0.022 |  | -0.141 | -0.492 | 0.211 | 0.433 |  |
| Flat | 0.039 | -0.362 | 0.440 | 0.849 |  | 0.330 | -0.443 | 1.102 | 0.403 |  |
| Dual (Ref) | **4.163** | - | - | - |  | **4.047** | - | - | - |  |
| Location of Incident |  |  |  |  |  |  |  |  |  |  |
| Racecourse | -0.084 | -0.428 | 0.261 | 0.634 |  | -0.557 | -1.609 | 0.495 | 0.299 |  |
| Other (Ref) | **4.000** | - | - | - |  | **4.047** | - | - | - |  |
| Race Incident |  |  |  |  |  |  |  |  |  |  |
| Race Incident | -0.066 | -0.390 | 0.259 | 0.691 |  | 0.115 | -0.706 | 0.936 | 0.783 |  |
| Other Riding Incident (Ref) | **3.983** | - | - | - |  | **4.047** | - | - | - |  |
| Race Type |  |  |  |  |  |  |  |  |  |  |
| Steeplechase | -0.010 | -0.348 | 0.328 | 0.953 |  | 0.367 | -0.268 | 1.001 | 0.257 |  |
| Hurdle | -0.171 | -0.523 | 0.181 | 0.342 |  | 0.348 | -0.299 | 0.995 | 0.292 |  |
| Flat | 0.147 | -0.263 | 0.558 | 0.482 |  | 0.314 | -0.382 | 1.010 | 0.376 |  |
| Non-Race Related (Ref) | **3.956** | - | - | - |  | **4.047** | - | - | - |  |
| Management Approach |  |  |  |  |  |  |  |  |  |  |
| Surgical | 0.591 | 0.307 | 0.874 | <0.001 |  | 0.472 | 0.165 | 0.779 | 0.003 |  |
| Conservative (Ref) | **3.799** | - | - | - |  | **4.047** | - | - | - |  |
| Displacement |  |  |  |  |  |  |  |  |  |  |
| Displaced | 0.406 | 0.072 | 0.740 | 0.017 |  | 0.323 | -0.004 | 0.650 | 0.053 |  |
| Undisplaced | 0.097 | -0.296 | 0.490 | 0.627 |  | 0.154 | -0.213 | 0.522 | 0.411 |  |
| Unknown (Ref) | **3.836** | - | - | - |  | **4.047** | - | - | - |  |
| Riding Experience (years)* | -0.098 | -0.200 | 0.004 | 0.058 |  | -0.142 | -0.359 | 0.076 | 0.202 |  |
| Career Races (no.)* | -0.049 | -0.148 | 0.050 | 0.332 |  | 0.347 | 0.016 | 0.678 | 0.040 |  |
| Races per Year (no.)* | -0.088 | -0.192 | 0.016 | 0.097 |  | -0.411 | -0.709 | -0.113 | 0.007 |  |

S1 Table 1. Log transformed results from the univariable and multivariable gamma generalised linear model.

Note: * = Grand mean centred and scaled. Ref = reference or referent group. Bold = intercept.
