# Supplementary material for: Factors associated with time to return to horse racing following a clavicle fracture in jockeys competing in Great Britain: A review and analysis of medical records
Source: PLoS One. 2025 Jan 23;20(1):e0317724. doi: 10.1371/journal.pone.0317724 (PMC11756748; doi:10.1371/journal.pone.0317724)
Supplement: S2 Table — Note: * = Grand mean centred and scaled. Ref = reference or referent group. (DOCX) [file pone.0317724.s002.docx]

S2, Table. Exponentiated results from the univariable and multivariable gamma generalised linear model.

|  | Univariable Model | | | |  | Multivariable Model | | | | |
| --- | --- | --- | --- | --- | --- | --- | --- | --- | --- | --- |
| Independent variable | e^β^ | Lower 95% CI | Upper 95% CI | P value |  | e^β^ | Lower 95% CI | Upper 95% CI | P value |  |
| Sex |  |  |  |  |  |  |  |  |  |  |
| Male | 0.84 | 0.61 | 1.16 | 0.285 |  | 1.16 | 0.83 | 1.62 | 0.382 |  |
| Female (Ref) | - | - | - | - |  | - | - | - | - |  |
| Licence Type |  |  |  |  |  |  |  |  |  |  |
| Amateur | 0.67 | 0.42 | 1.06 | 0.090 |  | 0.72 | 0.36 | 1.47 | 0.371 |  |
| Conditional | 0.56 | 0.35 | 0.90 | 0.017 |  | 0.66 | 0.32 | 1.33 | 0.243 |  |
| Professional | 0.62 | 0.38 | 1.01 | 0.056 |  | 0.76 | 0.38 | 1.54 | 0.451 |  |
| Apprentice (Ref) | - | - | - | - |  | - | - | - | - |  |
| Jockey Type |  |  |  |  |  |  |  |  |  |  |
| Jump | 0.69 | 0.51 | 0.95 | 0.022 |  | 0.87 | 0.61 | 1.23 | 0.433 |  |
| Flat | 1.04 | 0.70 | 1.55 | 0.849 |  | 1.39 | 0.64 | 3.01 | 0.403 |  |
| Dual (Ref) | - | - | - | - |  | - | - | - | - |  |
| Location of Incident |  |  |  |  |  |  |  |  |  |  |
| Racecourse | 0.92 | 0.65 | 1.30 | 0.634 |  | 0.57 | 0.20 | 1.64 | 0.299 |  |
| Other (Ref) | - | - | - | - |  | - | - | - | - |  |
| Race Incident |  |  |  |  |  |  |  |  |  |  |
| Race Incident | 0.94 | 0.68 | 1.30 | 0.691 |  | 1.12 | 0.49 | 2.55 | 0.783 |  |
| Other Riding Incident (Ref) | - | - | - | - |  | - | - | - | - |  |
| Race Type |  |  |  |  |  |  |  |  |  |  |
| Steeplechase | 0.99 | 0.71 | 1.39 | 0.953 |  | 1.44 | 0.76 | 2.72 | 0.257 |  |
| Hurdle | 0.84 | 0.59 | 1.20 | 0.342 |  | 1.42 | 0.74 | 2.70 | 0.292 |  |
| Flat | 1.16 | 0.77 | 1.75 | 0.482 |  | 1.37 | 0.68 | 2.75 | 0.376 |  |
| Non-Race Related (Ref) | - | - | - | - |  | - | - | - | - |  |
| Management Approach |  |  |  |  |  |  |  |  |  |  |
| Surgical | 1.81 | 1.36 | 2.40 | <0.001 |  | 1.60 | 1.18 | 2.18 | 0.003 |  |
| Conservative (Ref) | - | - | - | - |  | - | - | - | - |  |
| Displacement |  |  |  |  |  |  |  |  |  |  |
| Displaced | 1.50 | 1.07 | 2.10 | 0.017 |  | 1.38 | 1.00 | 1.92 | 0.053 |  |
| Undisplaced | 1.10 | 0.74 | 1.63 | 0.627 |  | 1.17 | 0.81 | 1.69 | 0.411 |  |
| Unknown (Ref) | - | - | - | - |  | - | - | - | - |  |
| Riding Experience (years)* | 0.91 | 0.82 | 1.00 | 0.058 |  | 0.87 | 0.70 | 1.08 | 0.202 |  |
| Career Races (no.)* | 0.95 | 0.86 | 1.05 | 0.332 |  | 1.41 | 1.02 | 1.97 | 0.040 |  |
| Races per Year (no.)* | 0.92 | 0.83 | 1.02 | 0.097 |  | 0.66 | 0.49 | 0.89 | 0.007 |  |

Note: * = Grand mean centred and scaled. Ref = reference or referent group.
